# Distributions of Usage and the Costs of Conventional Medicine and Traditional Chinese Medicine for Lung Cancer Patients in Taiwan

**DOI:** 10.1155/2013/984876

**Published:** 2013-07-16

**Authors:** Yueh-Hsiang Liao, Jaung-Geng Lin, Cheng-Chieh Lin, Tsai-Chung Li

**Affiliations:** ^1^School of Chinese Medicine, China Medical University, Taichung 40402, Taiwan; ^2^Department of Family Medicine, China Medical University Hospital, Taichung 40402, Taiwan; ^3^School of Medicine, College of Medicine, China Medical University, Taichung 40402, Taiwan; ^4^Department of Medical Research, China Medical University Hospital, Taichung 40402, Taiwan; ^5^Graduate Institute of Biostatistics, China Medical University, Taichung 40402, Taiwan; ^6^Department of Healthcare Administration, College of Health Science, Asia University, Taichung 41354, Taiwan

## Abstract

*Background*. This study aims to analyze the utilization patterns of patients with lung cancer stratified by surgery status. *Methods*. A retrospective cohort study was conducted from 1996 to 2010 by using the Longitudinal Health Insurance Database 2005. *Results*. Among the 7,677 lung cancer patients, 230 (31.17%) and 1,826 (26.32%) who have and have not undergone surgery have used TCM outpatient services, respectively. For lung cancer patients who have not undergone surgery, patients who are aged 70 years and older, males, occupational members, and farmers and fishermen are less likely to avail of TCM services. For lung cancer patients who have undergone surgery, the likelihood of TCM users is higher in residents who used TCM one year prior to lung cancer diagnosis and in patients with insurance amounts ranging from ≥NT$60,000. The total amount paid per visit for WM is higher than that for one year of TCM outpatient care before and after lung cancer diagnosis. *Conclusion*. The factors associated with TCM use varied according to surgery status. The costs of insurance covering TCM were consistently lower than those covering WM for lung cancer patients. These findings would be useful for health policy makers who are considering TCM and WM integration.

## 1. Introduction

### 1.1. Incidence and Mortality of Lung Cancer

Lung cancer is the leading cause of cancer-related mortality worldwide. Northern America, Eastern Asia, and Western Europe have the highest incidence of lung cancer, whereas the Eastern, Western, and Middle Africa have the lowest incidence of lung cancer. In particular, Hungary, French Polynesia, United States of America, Poland, and Serbia have the highest incidence rates of lung cancer in the world; their age-standardized incidence rates in 2008 were 52.0, 43.6, 42.1, 40.9, and 40.7 per 100,000 persons, respectively [1]. About 55% of lung cancer cases occur in less developed countries. Lung cancer accounts for more than 28% of the total deaths in Taiwan [2]. Lung cancer also ranks first as the most common cancer in both men and women, accounting for about 20% of all cancer deaths. In addition, the annual standardized mortality incidence increased from 22.7/100,000 in 1991 to 25.9/100,000 in 2009 [2]. Lung cancer accounts for more than 20% of all cancer deaths in Hong Kong and ranks first as the most common cancer in the country [3].

### 1.2. Use of Complementary and Alternative Medicine (CAM) Worldwide

The use of CAM has gained worldwide popularity. According to a survey across a number of European countries, the top three motives for using CAM in cancer patients include directly fighting the disease with alternative therapy for decreasing the tumor, increasing the ability of the body to fight the cancer, and improving the physical well-being of the patient [4]. CAM, which is commonly used with conventional medicine for cancer care, is covered by the National Health Insurance (NHI) Program of Taiwan [5–8]. According to the 2007 National Health Interview Survey, the prevalence of CAM use was 42% in Asian Americans and 38% in American adults [9]. The percentage of CAM use was 23.6% for lung cancer patients, which is slightly higher than that for head and neck cancer (22.7%), but lower than that for pancreatic cancer patients (56.3%) [4].

### 1.3. Use of Traditional Chinese Medicine (TCM) in Asian Countries

TCM is one of the most popular forms of CAM worldwide. Cancer patients use TCM because they believe that TCM is a self-help cultural process and because they believe that conventional therapy has adverse effects and individualized and tailored prescriptions are important [10]. TCM is commonly used with conventional medicine and has entered the mainstream society and culture. TCM is even covered by the NHI program of Taiwan. In addition, TCM plays an active role in the modern health care system of Chinese and East Asian societies. One important feature of the NHI program is the coverage of both biomedicine (WM) and TCM. By 2003, more than 99% of the 23 million residents of Taiwan had been covered by the NHI program after the implementation of this universal health insurance plan. Thus, TCM has a higher level of accessibility because of less financial barrier. A study on the determinants of TCM and acupuncture utilization of patients with cervical, breast, lung, liver, or colorectal cancers in Taiwan has shown that the prevalence of TCM and acupuncture use for lung cancer patients is 16.03% and 2.53%, respectively [11].

### 1.4. Therapeutic Effect of TCM against Non-Small-Cell Lung Cancer (NSCLC) in Previous Studies

TCM is commonly used in combination with chemotherapy or radiotherapy in treating patients with unresectable NSCLC [12–16]. The effect of treatment with TCM has been focused on the stimulation of the host immune response, thus activating a cytotoxic response against NSCLC. A systematic review including publications in 11 electronic databases and 24 trials has evaluated the efficacy of Chinese herbal medicine (CHM) combined with conventional chemotherapy (CT) in treatment of advance NSCLC [17]. Their findings indicated that the most five commonly used herbs were *Radix Adenophorae, Radix Ophiopogonis, Radix Glycyrrhizae, *and* Poria*. In addition, CHM as an adjuvant therapy can reduce CT toxicity in terms of reduction of nausea and vomiting, decrease of hemoglobin, inhibition of white blood cells, and decrease of platelet, prolong one-year survival rate, enhance immediate tumor response, and improve Karnofsky performance score in advanced NSCLC patients [17]. Another systematic review on oral CHM trials involving 862 patients with NSCLC showed that TCM plus chemotherapy improves the quality of life, tumor response, and survival rate of patients, as well as alleviate the symptoms experienced by patients [12]. The systematic review also demonstrated that aidi injection, a type of TCM, combined with cobalt-60 or navelbine and platinol has adjuvant therapeutic effects in improving the response rate, bone marrow hematopoietic function, and quality of life but not the survival rate of patients [13]. In a randomized control, TCM by stages plus chemotherapy significantly increases the survival and quality of life of patients with advanced NSCLC [14]. *Astragalus mongholicus, Gynostemma pentaphyllum, Ganoderma lucidum, Ligustrum lucidum, Atractylodes, Coptis chinensis, *and* Coptis chinensis* are the most commonly used ingredients that have demonstrated oncologic and immunologic pharmacology for lung cancer [14, 15]. These ingredients either inhibit lung adenocarcinoma cell migration and invasion or have an anticancer effect by inhibiting human cancer cell growth and inducing apoptosis [14–16]. The possible anticancer mechanisms of these ingredients include cell cycle arrest following apoptotic death by numerous and competing degenerative pathways [15].

### 1.5. How the Current Study Helps Resolve Uncertainties regarding TCM Use

Several studies have explored the prevalence of TCM use. However, these studies have been conducted either in general populations [18–23], in a single clinical setting [24], or for acupuncture use only [25]. Two studies have explored TCM use in Taiwan in 1996–2001 [26] and in 1997–2003 [27] in a general population by using complete NHI datasets for TCM. Two recent studies have explored TCM use among patients with prostate [28] and liver cancers [5]. However, TCM use among patients with lung cancer has not yet been reported and the impact of surgery on TCM use has not yet been explored. This study aims to compare the differences between the characteristics, types of care provider, existing diseases, and expenditures for outpatient services of TCM and non-TCM users with lung cancer enrolled in the NHI program. This study, which is stratified by the surgery status of lung cancer patients in Taiwan from 1996 to 2010, utilized a population-based random sample of one million insured patients.

## 2. Methods

### 2.1. Data Sources

This study used NHI Research Database (NHIRD) claim datasets from the NHI program of Taiwan. The NHI program was initiated in Taiwan in March 1995 and covers approximately 99% of the 23.74 million Taiwanese residents [29]. The national government-run Bureau of National Health Insurance (BNHI) had contracts with 97% of the hospitals and 92% of the clinics all over the nation. The NHIRD provides registration and claim datasets from a random sample of one million beneficiaries for research use. The Longitudinal Health Insurance Database 2005 (LHID2005) contains all the ambulatory and inpatient claim data of one million beneficiaries who were randomly sampled from the entire enrollees in the NHI and was released in 2007. Similar distributions of beneficiary age and gender in the LHID2005 and the original NHI database were observed. The registration and claim datasets from the LHID2005 for the years 1996 to 2010 were used in the current study. The LHID2005 database contains comprehensive information, such as the demographic data, dates of clinical visits, diagnostic codes, details of prescriptions, and expenditure amounts of beneficiaries. The data for registered beneficiaries, expenditures of ambulatory care visits, and inpatient admission were obtained for analysis. Every individual has a unique personal identification number (PIN). The data on patient identities and institutions were scrambled cryptographically by the NHIRD to protect the privacy of the beneficiaries. All datasets can be interlinked by using an individual's PIN. The BNHI conducts an expert review of random samples of every 50 to 100 ambulatory and inpatient claims in each hospital and clinic quarterly; several penalties are given to hospitals and clinics that generate false reports of diagnosis [30]. The International Classification of Disease, Ninth Revision, Clinical Modification (ICD-9-CM) was used in disease diagnosis.

### 2.2. Study Subjects

A retrospective cohort study for patients with lung cancer was conducted. An individual with lung cancer had to have at least three ambulatory claims or at least one inpatient claim with diagnosis of ICD-9-CM code 162 from 1997 to 2009, in which the first diagnosis date is the index date. A total of 7,677 lung cancer patients utilized ambulatory or inpatient care during the period. The surgery status of the patients was identified by using procedures including wedge resection (32.29), thoracoscopic wedge resection (32.20), segmental resection (32.39), thoracoscopic segmental section (32.30), lobectomy (32.49), thoracoscopic lobectomy (32.41), pneumonectomy (32.59), or thoracoscopic pneumonectomy (32.50).

### 2.3. Variables for Expenditures and Coexisting Diseases

The NHI covered TCM outpatient care, but not TCM inpatient care. Therefore, only TCM and WM ambulatory care were analyzed in this study. The outpatient datasets contained encounter forms based on dates of visit, patient gender and date of birth, medical facility visited, department visited, prescribing physician, dispensing pharmacist, three items from the ICD-9-CM codes, primary procedure (e.g., drug or diagnostic procedure), type of copayment, and billed and paid amounts. All outpatient visits within one year, particularly before and after the index date, were analyzed for expenditure and coexisting diseases.

### 2.4. Sociodemographic Factors and Urbanization Levels of Residential Area

The sociodemographic factors studied included age, gender, insurance premium, and insured unit. Age was categorized into four levels: <50, 50–59, 60–69, and ≥70 (in years). Amount of insurance premium was categorized into four levels: <20,000, 20,000–39,999, 40,000–59,999, and ≥60,000. The insurance premium amount of an individual was determined by his/her work salary. The residential areas of study subjects consisted of 6 areas: Northern, Taipei, Central, Southern, Eastern, and Kao-Ping areas. The Kao-Ping area is located at southwestern Taiwan, consisting of Kaohsiung City, Kaohsiung County, and Ping-Tung County. The urbanization level of 365 Taiwan townships was classified into seven levels according to the method developed by Lin et al. [31]. The seven levels of urbanization were high-density urban area, medium-density urban area, newly developed area, general area, aging society area, rural area, and nondeveloped area (seclusion area). The indicators used in developing the township stratification for urbanization level included the population density (people/km^2^), population ratio of people with an educational level of college or above, population ratio of elder people over 65 years old, population ratio of agricultural workers, and the number of physicians per 100,000 people, among others. The insured unit consisted of governmental department and public school, private enterprise, occupational member, farmers and fishermen, and low-income households and veterans.

### 2.5. Statistical Analysis

The mean and 95% confidence interval (CI) were reported for the continuous variables, whereas the number, percentage, and 95% CI were reported for the categorical variables. The chi-square test or the two-sample *t*-test was used to compare the differences in proportions and means of the variables. The adjusted odd ratios (ORs) of TCM use for each factor were estimated by multivariate logistic regression analysis. Cox proportional hazard models were used to evaluate the association between TCM use and 5-year mortality. We calculated hazard ratios and their 95% confidence intervals (CIs) by adjusting age, gender, and multiple variables. All analyses were stratified by surgery status to examine the status of one of the major lung cancer treatments on TCM use and pattern. All *P* values were reported from two-sided tests, in which the level of statistical significance was set at 0.05. All analyses were performed by using SAS version 9.3 (SAS Institute Inc., Cary, NC, USA).

## 3. Results

### 3.1. Factors Associated with TCM Use

Among the 7,677 lung cancer patients, 1,826 (26.32%) lung cancer patients who have not undergone surgery have availed of TCM outpatient services, whereas 230 (31.17%) lung cancer patients who have undergone surgery have availed of TCM outpatient services. Among the lung cancer patients who have not undergone surgery, participants aged 70 years and older (OR = 0.60, 95% confidence interval, CI: 0.49–0.73), males (0.86, 0.75–0.98), occupational members (0.69, 0.51–0.93), and farmers and fishermen (0.72, 0.53–0.97) are less likely to avail of TCM services. By contrast, patients who are TCM users one year prior to lung cancer diagnosis (4.55, 4.00–5.17), who have insurance amounts ranging from NT$20,000 to NT$39,999 (1.34, 1.12–1.60), who are residents of urban levels 2 (1.35, 1.03–1.77) and 4 (1.47, 1.14–1.91) and Central (1.55, 1.21–1.97) and Kao-Ping areas (1.32, 1.02–1.71) are more likely to avail of TCM services. Among the lung cancer patients who have undergone surgery, the likelihood of TCM users is higher in residents who used TCM one year prior to lung cancer diagnosis (4.07, 2.74–6.04) and in patients with insurance amount ranging from ≥NT$60,000 (3.18, 1.18–8.56) (Table 1).

### 3.2. Medical Institutes

For patients who have not undergone surgery, most WM and TCM outpatient services one year after lung cancer diagnosis were provided by private hospitals (43.76% for WM; 36.07% for TCM) and private clinics (26.75% for WM; 40.46% for TCM) (Table 2). For patients who have undergone surgery, most WM outpatient services one year after lung cancer diagnosis were provided by private hospitals (45.65%), followed by public hospitals (32.14%). Most TCM outpatient services one year after lung cancer diagnosis were provided by private hospitals (45.22%), followed by private clinics (29.91%). The outpatient services one year before lung cancer diagnosis of patients who have and have not undergone surgery had similar patterns: most WM and TCM outpatient services were provided by private clinics, followed by private hospitals.

### 3.3. Coexisting Diseases

The diagnoses in all ambulatory claim data were recorded in the ICD-9-CM format. One year after lung cancer diagnosis, lung cancer was the top disease code for WM and TCM patients who have and have not undergone surgery. Except in TCM users who have undergone surgery, the three most frequently recorded coexisting diseases in patients were essential hypertension, diabetes mellitus, and acute upper respiratory infection. For TCM users who have undergone surgery, the three most frequently recorded coexisting diseases were essential hypertension, diabetes mellitus, and general symptoms. The most frequently recorded coexisting diseases of patients one year before lung cancer diagnosis were acute upper respiratory infection, followed by essential hypertension, diabetes mellitus, and general symptoms (see Table 3).

### 3.4. Expenditures

Table 4 shows the expenditure details of the lung cancer patients. For the patients who have not undergone surgery, the WM outpatient services accounted for 64.40% of all outpatient visits and 71.15% of the total expenditures for one-year outpatient care after cancer diagnosis, whereas the WM outpatient services accounted for 70.12% of the visits and 66.67% of the expenditures for one-year outpatient care before cancer diagnosis. For the patients who have undergone surgery, the WM outpatient services accounted for 61.64% of all outpatient visits and 43.21% of the total expenditures for one-year outpatient care after cancer diagnosis, whereas the WM outpatient services accounted for 63.05% of the visits and 48.25% of the expenditures for one-year outpatient care before cancer diagnosis. The cost of consultation, treatment, medical supplies, drugs, and visitations of TCM nonusers who have not undergone surgery were much higher than those of TCM users who undergone one-year outpatient care before and after cancer diagnosis. The average total expenditure of TCM nonusers was NT$2990.92 (US$98.39) per visit after cancer diagnosis and NT$1257.98 (US$41.38) per visit before cancer diagnosis. The average total expenditure of TCM users was NT$2193.82 (US$72.17) per visit after cancer diagnosis and NT$982.16 (US$32.31) per visit after cancer diagnosis (US$ 1 = NT$30.4 in 2010). For patients who have undergone surgery, the fee for consultation, treatment, and medical supplies, as well as the cost for the total amount per visit of WM, was also much higher than those of TCM for one-year outpatient care before and after cancer diagnosis. The average total expenditure of TCM nonusers was NT$3104.64 (US$102.13) per visit after cancer diagnosis and NT$1270.36 (US$41.79) per visit before cancer diagnosis. The average total expenditure of TCM users was NT$2783.77 (US$91.57) per visit after cancer diagnosis and NT$1121.71 (US$36.90) per visit before cancer diagnosis.

### 3.5. Overall Mortality

Table 5 shows the univariate and multivariate Cox's analyses of TCM use for overall mortality stratified by surgery status. We found there was no statistical difference in overall mortality between TCM and non-TCM users with surgery. On the contrary, TCM users were associated with lower mortality than non-TCM users in patients without surgery (adjusted hazard ratio for mortality: 0.64; 95% CI: 0.55–0.76).

## 4. Discussion

This study is the first large-scale survey in the literature that focuses on TCM use for lung cancer patients who undergo one-year outpatient care before and after lung cancer diagnosis. In this study, for patients who have not undergone surgery, the proportions of TCM outpatient visits increased from 29.88% before lung cancer diagnosis to 35.60% after lung cancer diagnosis. On the contrary, the proportions of TCM outpatient expenditures decreased from 33.33% to 28.85%. For patients who have undergone surgery, the proportions of TCM outpatient visits and expenditures increased from 36.95% and 51.75% before lung cancer diagnosis to 38.36% and 56.79% after lung cancer diagnosis, respectively. The possible explanation for this finding is that TCM use is associated with longer survival in lung cancer patients with surgery as found in our study.

Among the lung cancer patients who have not undergone surgery, TCM use was lower among ≥70 year old patients, males, occupational members, farmers, and fishermen. On the contrary, the percent of TCM use was higher among those who reside in the regions of central Taiwan. These findings are similar to those of a previous study on patients with mild diseases or prostate cancer [5, 26–28]. The overall prevalence of TCM utilization in the current study is higher than that reported by Lin et al. for patients with prostate cancer (26.78% versus 2.6%) [28] and that by our previous results for patients with liver cancer (26.78% versus 19.50%) [5]. Lin et al. considered only the outpatient visits specific for prostate cancer. By contrast, the current study considered all outpatient visits. Therefore, the current study reports a higher prevalence of TCM use compared with that of Lin et al. On the basis of our previous work on liver cancer, a slightly higher prevalence of TCM utilization was shown in patients with lung cancer compared with patients with liver cancer. The possible explanation for this higher prevalence is that patients with lung cancer were associated with higher prevalence of respiratory disease, and the Chinese are more likely to seek TCM to relieve symptoms caused by respiratory diseases. In this study, we further consider the effects of the surgery status of TCM users prior to cancer diagnosis on their TCM use after cancer diagnosis. Results show that surgery status did not exert a significant effect on TCM utilization. Prior TCM use before cancer diagnosis is shown to be the most significant factor for TCM users who have not undergone and have undergone surgery. The significant factor of prior TCM use before cancer diagnosis, a socio-cultural characteristics of individuals that exist prior to their illness, corresponds to the predisposing factor that proposed by Andersen's health behavior model [32].

On the basis of the ICD-9-CM codes, and with the exception of the malignant lung neoplasm, we found that acute upper respiratory infections, diabetes mellitus, and essential hypertension were the primary indications in TCM users and nonusers. In TCM users who have and have not undergone surgery, the other primary indications were symptoms involving the respiratory system and other chest symptoms. Pulmonary tuberculosis was shown to be another indication for TCM use. These findings on disease pattern of health care use may be explained by the fact that patients seek TCM to relieve respiratory symptoms.

Using the prevalence of surgery and difference in total costs for each TCM and WM user per year from our study, and number of lung cancer incidence cases in 2008 reported by Department of Health, Taiwan, we estimated that TCM use would save at least 7.1 millions per year. Given that costs for TCM and WM use per session in other developed countries are much higher than those in Taiwan, we believe that TCM use would potentially save much more costs in other developed countries.

This study is the first large-scale study of TCM use in Taiwanese society, in which a million residents were randomly selected from the 23 million population of Taiwan. The NHI database has high comprehensiveness because the NHI covers more than 99% of the population of Taiwan and 93% of the medical institutes. Previous studies of health care utilization in cancer patients did not consider the status of TCM use prior to cancer diagnosis. Several previous studies on TCM/CAM use have been conducted via telephone interviews, self-administered interviews, household interviews, and hospital- and clinical-based survey. These studies usually had limited sample sizes and were conducted in countries in which TCM/CAM was not covered by insurance. Thus, the pattern and characteristics of TCM/CAM use may be affected by the socioeconomic status of individuals.

This study has several limitations. First, several herbal medicines were not covered by NHI, and visits at clinics not in contract with the BNHI were not considered in this study. About 10% of the TCM clinics do not have contracts with NHI because of the low insurance amount given by NHI. In addition, cancer patients need rare and expensive Chinese herbal medicine, which is not covered by NHI. The NHIRD data may lead to an underestimation of TCM costs. Second, the data for the clinical stages and types of lung cancer were not available for the study; thus, TCM use and costs stratified by these factors cannot be explored. We also observed that the pattern of TCM use varied according to surgery status. However, we did not observe a significant correlation of surgery and other covariates because of limited sample size of TCM users who have undergone surgery. Thus, we described the utilization pattern of TCM according to surgery status.

## 5. Conclusions

Our study reports the prevalence and pattern of TCM use under NHI, a comprehensive and universal health insurance program in Taiwan. The NHI program covers both conventional WM and TCM services. The prevalence of TCM use among lung cancer patients who have and have not undergone surgery is similar. The factors associated with TCM use varied according to surgery status. The total costs of insurance-covered TCM were lower than those of WM for both patients who have and have not undergone surgery. This study provides information about the frequency of TCM use and the coexisting diseases treated by WM and TCM in lung cancer patients according to surgery status. The results of this study would be useful for health policy makers and for researchers considering TCM and WM integration.

## Figures and Tables

**Table 1:** Sociodemographic factors of patients with lung cancer.

Characteristic	Nonsurgery (*N* = 6,939)			Surgery (*N* = 738)			*P* value for interaction
	TCM nonusers	TCM users	Adjusted^a^ OR (95% CI)	TCM nonusers	TCM users	Adjusted^a^ OR (95% CI)	
Patient no.	5113	1826		508	230		
Age	66.95 ± 14.95	61.59 ± 14.66		61.02 ± 12.82	59.85 ± 12.56		0.45
<50	789 (15.43)	412 (22.56)	1.00	88 (17.32)	47 (20.43)	1.00	
50s	784 (15.33)	339 (18.57)	0.85 (0.69–1.05)	132 (25.98)	72 (31.30)	1.24 (0.72–2.13)	
60s	1186 (23.20)	469 (25.68)	0.85 (0.70–1.04)	148 (29.13)	59 (25.65)	0.88 (0.48–1.61)	
≥70s	2354 (46.04)	606 (33.19)	0.60 (0.49–0.73)^+^	140 (27.56)	52 (22.61)	0.68 (0.35–1.31)	
Gender							0.43
Female	1855 (36.28)	796 (43.59)	1.00	195 (38.39)	115 (50.00)	1.00	
Male	3258 (63.72)	1030 (56.41)	0.86 (0.75–0.98)*	313 (61.61)	115 (50.00)	0.69 (0.46–1.04)	
TCM use one year prior to lung cancer diagnosis							0.45
TCM nonusers	4024 (78.70)	799 (43.76)	1.00	397 (78.15)	111 (48.26)	1.00	
TCM users	1089 (21.30)	1027 (56.24)	4.55 (4.00–5.17)^+^	111 (21.85)	119 (51.74)	4.07 (2.74–6.04)^+^	
Insured amount (NT$/month)							0.53
<20,000	3292 (64.38)	1079 (59.09)	—	297 (58.46)	127 (55.22)	1.00	
20,000–39,999	1476 (28.87)	527 (31.33)	1.34 (1.12–1.60)^#^	159 (31.30)	67 (29.13)	1.46 (0.83–2.58)	
40,000–59,999	218 (4.26)	113 (6.19)	1.23 (0.92–1.66)	41 (8.07)	22 (9.57)	1.93 (0.95–3.91)	
≥60,000	127 (2.48)	62 (3.40)	1.10 (0.75–1.61)	11 (2.17)	14 (6.09)	3.18 (1.18–8.56)*	
Urban level							0.67
1	1217 (25.80)	478 (28.901)	1.00	141 (29.87)	74 (36.45)	1.00	
2	1298 (27.52)	407 (24.61)	1.35 (1.03–1.77)*	128 (27.12)	51 (25.12)	2.05 (0.83–5.04)	
3	704 (14.92)	293 (17.71)	1.08 (0.84–1.40)	76 (16.10)	31 (15.27)	1.70 (0.71–4.08)	
4	818 (17.34)	300 (18.14)	1.47 (1.14–1.91)^#^	77 (16.31)	28 (13.79)	1.28 (0.54–3.05)	
≥5	680 (14.42)	176 (10.64)	1.22 (0.96–1.55)	50 (10.59)	19 (9.36)	1.49 (0.66–3.32)	
Residential area							0.79
Northern	592 (12.27)	177 (10.45)	1.00	50 (10.44)	19 (9.18)	1.00	
Taipei	1655 (34.31)	543 (32.05)	1.09 (0.85–1.39)	197 (41.13)	80 (38.65)	0.82 (0.38–1.78)	
Central	886 (18.37)	434 (25.62)	1.55 (1.21–1.97)^+^	71 (14.82)	44 (21.26)	1.44 (0.64–3.21)	
Southern	799 (16.57)	246 (14.46)	1.12 (0.86–1.46)	86 (17.95)	32 (15.46)	1.02 (0.45–2.33)	
Eastern	143 (2.96)	39 (2.30)	1.10 (0.70–1.73)	6 (1.25)	2 (0.97)	1.49 (0.21–10.54)	
Kao-Ping	748 (15.51)	256 (15.11)	1.32 (1.02–1.71)*	69 (14.41)	30 (14.49)	0.96 (0.41–2.23)	
Insured unit							0.96
Government, school employees	310 (6.81)	134 (8.10)	1.00	44 (9.57)	17 (8.67)	1.00	
Private enterprise employees	914 (20.09)	436 (26.34)	1.00 (0.77–1.29)	123 (26.74)	61 (31.12)	1.13 (0.55–2.32)	
Occupational member	600 (13.19)	235 (14.20)	0.69 (0.51–0.93)*	88 (19.13)	33 (16.84)	0.75 (0.33–1.74)	
Farmers, fishermen	1351 (29.69)	404 (24.41)	0.72 (0.53–0.97)*	101 (21.96)	40 (20.41)	1.45 (0.60–3.52)	
Low-income households and veterans, other regional	1375 (30.22)	446 (26.95)	0.98 (0.75–1.28)	104 (22.61)	45 (22.96)	1.88 (0.84–4.24)	

TCM: traditional Chinese medicine; OR: odds ratio; CI: confidence interval. *<0.05; ^#^<0.01; ^+^<0.001; ^a^adjusted ORs were from the model considering age, gender, visit one year ago, insured amount, residential area, and insured unit.

**Table 2:** Lung cancer outpatient service providers during the period 1996–2010.

Outpatient service providers	Nonsurgery (*N* = 6,939)					Surgery (*N* = 738)				
	TCM nonusers (*N* = 5,113)		TCM users (*N* = 1,826)		*P* value for *χ* ^2^	TCM nonusers (*N* = 508)		TCM users (*N* = 230)		*P* value for *χ* ^2^
	Visits	Percentage (95% CI)	Visits	Percentage (95% CI)		Visits	Percentage (95% CI)	Visits	Percentage (95% CI)	
Visits one year after lung cancer diagnosis										
Type of providers					<0.001					<0.001
Public hospitals	37,079	26.15 (25.92, 26.38)	15,825	20.19 (19.91, 20.47)		5,832	32.14 (31.46, 32.82)	2,616	23.17 (22.39, 23.95)	
Public Chinese medicine hospitals	0	—	129	0.16 (0.14, 0.19)		0	—	6	0.05 (0.01, 0.1)	
Private hospitals	62,051	43.76 (43.5, 44.02)	28,271	36.07 (35.74, 36.41)		8,284	45.65 (44.92, 46.37)	5,106	45.22 (44.3, 46.14)	
Private Chinese medicine hospitals	0	—	577	0.74 (0.68, 0.8)		0	—	22	0.19 (0.11, 0.28)	
Public clinics	4,325	3.05 (2.96, 3.14)	1,712	2.18 (2.08, 2.29)		419	2.31 (2.09, 2.53)	141	1.25 (1.04, 1.45)	
Private clinics	37,930	26.75 (26.52, 26.98)	31,712	40.46 (40.12, 40.81)		3,594	19.8 (19.22, 20.38)	3,377	29.91 (29.06, 30.75)	
Other medicine service providers	407	0.29 (0.26, 0.31)	146	0.19 (0.16, 0.22)		19	0.10 (0.06, 0.15)	24	0.21 (0.13, 0.30)	

Total	141,792	100	78,372	100		18,148	100	11,292	100	

Visits one year prior to lung cancer diagnosis										
Type of providers					<0.001					<0.001
Public hospitals	22,613	17.02 (16.81, 17.22)	8,176	14.44 (14.15, 14.73)		2,456	19.16 (18.48, 19.84)	1,138	15.15 (14.34, 15.96)	
Public Chinese medicine hospitals	38	0.03 (0.02, 0.04)	85	0.15 (0.12, 0.18)		6	0.05 (0.01, 0.08)	0	—	
Private hospitals	42,914	32.29 (32.04, 32.55)	15,163	26.78 (26.41, 27.14)		4,227	32.98 (32.17, 33.79)	2,334	31.07 (30.02, 32.12)	
Private Chinese medicine hospitals	206	0.16 (0.13, 0.18)	491	0.87 (0.79, 0.94)		39	0.30 (0.21, 0.4)	14	0.19 (0.09, 0.28)	
Public clinics	5,826	4.38 (4.27, 4.49)	1,929	3.41 (3.26, 3.56)		579	4.52 (4.16, 4.88)	202	2.69 (2.32, 3.05)	
Private clinics	60,971	45.88 (45.61, 46.15)	30,716	54.24 (53.83, 54.65)		5,493	42.86 (42, 43.71)	3,804	50.64 (49.51, 51.77)	
Other medicine service providers	318	0.24 (0.21, 0.27)	71	0.13 (0.10, 0.15)		17	0.13 (0.07, 0.2)	20	0.27 (0.15, 0.38)	

Total	132,886	100	56,631	100		12,817	100	7,512	100	

TCM: traditional Chinese medicine; CI: confidence interval.

**Table 3:** Top 5 disease codes among lung cancer patients during the years 1996–2010 for all outpatient visits one year before and after lung cancer diagnosis.

Ranking	Nonsurgery (*N* = 6,939)						Surgery (*N* = 738)					
	TCM nonusers (*N* = 5,113)			TCM users (*N* = 1,826)			TCM nonusers (*N* = 508)			TCM users (*N* = 230)		
	Disease (code)	No.	Percentage (95% CI)	Disease (code)	No.	Percentage (95% CI)	Disease (code)	No.	Percentage (95% CI)	Disease (code)	No.	Percentage (95% CI)
Outpatient visits one year after lung cancer diagnosis												
	Total visits = 141,792			Total visits = 78,372			Total visits = 18,148			Total visits = 11,292		
1	Lung cancer (162)	41,431	29.22 (28.98, 29.46)	Lung cancer (162)	16,308	20.81 (20.52, 21.09)	Lung cancer (162)	8,117	44.73 (44.00, 45.45)	Lung cancer (162)	4,674	41.39 (40.48, 42.3)
2	Essential hypertension (401)	11,917	8.40 (8.26, 8.55)	Acute upper respiratory infections (465)	4,989	6.37 (6.19, 6.54)	Diabetes mellitus (250)	1,150	6.34 (5.98, 6.69)	Essential hypertension (401)	661	5.85 (5.42, 6.29)
3	Diabetes mellitus (250)	11,480	8.10 (7.95, 8.24)	Essential hypertension (401)	4,692	5.99 (5.82, 6.15)	Essential hypertension (401)	1,135	6.25 (5.9, 6.61)	Diabetes mellitus (250)	658	5.83 (5.40, 6.26)
4	Acute upper respiratory infections (465)	7,968	5.62 (5.50, 5.74)	Diabetes mellitus (250)	4,450	5.68 (5.52, 5.84)	Acute upper respiratory infections (465)	798	4.40 (4.10, 4.70)	General symptoms (780)	560	4.96 (4.56, 5.36)
5	General symptoms (780)	6,652	4.69 (4.58, 4.80)	General symptoms (780)	3,526	4.50 (4.35, 4.64)	Symptoms involving respiratory system and other chest symptoms (786)	736	4.06 (3.77, 4.34)	Acute upper respiratory infections (465)	487	4.31 (3.94, 4.69)

Outpatient visits one years before lung cancer diagnosis												
	Total visits = 132,886			Total visits = 56,631			Total visits = 12,817			Total visits = 7,512		
1	Acute upper respiratory infections (465)	10,986	8.27 (8.12, 8.42)	Acute upper respiratory infections (465)	7,093	12.52 (12.25, 12.8)	Acute upper respiratory infections (465)	1,182	9.22 (8.72, 9.72)	Acute upper respiratory infections (465)	643	8.56 (7.93, 9.19)
2	Essential hypertension (401)	10,845	8.16 (8.01, 8.31)	Essential hypertension (401)	4,478	7.91 (7.69, 8.13)	Essential hypertension (401)	903	7.05 (6.60, 7.49)	Diabetes mellitus (250)	624	8.31 (7.68, 8.93)
3	Diabetes mellitus (250)	9,095	6.84 (6.71, 6.98)	Diabetes mellitus (250)	3,958	6.99 (6.78, 7.20)	Diabetes mellitus (250)	811	6.33 (5.91, 6.75)	Essential hypertension (401)	407	5.42 (4.91, 5.93)
4	General symptoms (780)	5,586	4.20 (4.10, 4.31)	General symptoms (780)	3,909	6.90 (6.69, 7.11)	General symptoms (780)	516	4.03 (3.69, 4.37)	General symptoms (780)	367	4.89 (4.40, 5.37)
5	Acute bronchitis and bronchiolitis (466)	4,747	3.57 (3.47, 3.67)	Symptoms involving respiratory system and other chest symptoms (786)	3,029	5.35 (5.16, 5.53)	Hypertensive heart disease (402)	432	3.37 (3.06, 3.68)	Acute bronchitis and bronchiolitis (466)	351	4.67 (4.20, 5.15)

**Table 4:** Expenditures for outpatient services for lung cancer patients (NT$) during the period 1996–2010 one year before and after lung cancer diagnosis.

Characteristic	Non-surgery (*N* = 6,939)							Surgery (*N* = 738)						*t* value
	TCM nonusers (*N* = 5,113)			TCM users (*N* = 1,826)			*t* value	TCM nonusers (*N* = 508)			TCM users (*N* = 230)			
	Total	Percentage (95% CI)	Average (95% CI)	Total	Percentage (95% CI)	Average (95% CI)		Total	Percentage (95% CI)	Average (95% CI)	Total	Percentage (95% CI)	Average (95% CI)	
*Outpatient visits one year after lung cancer diagnosis *	141,792			78,372				18,148			11,292			
Fees for consultation, treatment, and medical supply	181,842,461	42.88 (42.87, 42.88)	1282.46 (1249.09, 1315.83)	71,784,259	41.75 (41.74, 41.76)	915.94 (878.61, 953.28)	14.35***	29,957,288	53.17 (53.16, 53.18)	1650.72 (1538.08, 1763.36)	14,166,877	45.07 (45.05, 45.09)	1254.59 (1137.20, 1371.98)	4.77***
Diagnosis fee	30,666,528	7.23 (7.23, 7.23)	216.28 (215.63, 216.93)	17,116,958	9.96 (9.95, 9.96)	218.41 (217.67, 219.14)	−4.26***	3,764,169	6.68 (6.67, 6.69)	207.42 (205.82, 209.01)	2,437,653	7.75 (7.75, 7.76)	215.87 (213.89, 217.86)	−6.51***
Drug fee	211,579,372	49.89 (49.89, 49.90)	1492.18 (1462.74, 1521.62)	83,032,934	48.29 (48.29, 48.30)	1059.47 (1026.91, 1092.03)	19.32***	22,621,500	40.15 (40.14, 40.16)	1246.50 (1180.61, 1312.39)	14,829,824	47.18 (47.16, 47.19)	1313.30 (1219.56, 1407.04)	−1.14
Total amount	424,088,361	100	2990.92 (2945.98, 3035.86)	171,934,151	100	2193.82 (2143.54, 2244.10)	23.17***	56,342,957	100	3104.64 (2973.91, 3235.37)	31,434,354	100	2783.77 (2631.91, 2935.63)	3.14**

*Outpatient visits one year before lung cancer diagnosis *	132,886			56,631				12,817			7,512			
Fees for consultation, treatment, and medical supply	82,399,038	49.29 (49.28, 49.3)	620.07 (600.21, 639.94)	23,736,072	42.67 (42.66, 42.69)	419.14 (399.76, 438.51)	14.19***	8,969,651	55.09 (55.06, 55.11)	699.82 (633.15, 766.49)	3,851,397	45.71 (45.67, 45.74)	512.7 (474.3, 551.1)	4.77***
Diagnosis fee	29,313,743	17.54 (17.53, 17.54)	220.59 (220.01, 221.18)	12,468,308	22.42 (22.41, 22.43)	220.17 (219.39, 220.94)	0.86	2,752,677	16.91 (16.89, 16.92)	214.77 (212.98, 216.55)	1,661,546	19.72 (19.69, 19.75)	221.19 (218.79, 223.59)	−4.20***
Drug fee	55,455,774	33.17 (33.17, 33.18)	417.32 (409.28, 425.36)	19,416,544	34.91 (34.90, 34.92)	342.86 (329.2, 356.52)	9.20***	4,559,885	28.01 (27.98, 28.03)	355.77 (339.48, 372.06)	2,913,321	34.57 (34.54, 34.61)	387.82 (354.65, 421)	−1.70
Total amount	167,168,555	100	1257.98 (1236.48, 1279.48)	55,620,684	100	982.16 (958.35, 1005.97)	16.85***	16,282,213	100	1270.36 (1202.13, 1338.59)	8,426,264	100	1121.71 (1069.67, 1173.75)	3.40***

TCM: traditional Chinese medicine; *P < 0.05; **P < 0.01; ***P < 0.001; CI: confidence interval.

**Table 5:** Univariate and multivariate Cox's analyses of TCM use for overall mortality stratified by surgery status. (*n* = 7,677).

	*N *	Person-years	Cases	IR	Mortality	
					Age & gender adjusted HR (95% CI)	Adjusted^a^ HR (95% CI)
*Patients with surgery *						
TCM users						
No	508	2810.47	68	24.19	1.00	1.00
Yes	230	1090.53	29	26.59	1.06 (0.68–1.64)	0.95 (0.56–1.60)
*Patients without surgery *						
TCM users						
No	5113	25551.53	1184	46.34	1.00	1.00
Yes	1826	10908.33	272	24.94	0.67*** (0.58–0.76)	0.64*** (0.55–0.76)

*P < 0.05; **P < 0.01; ***P < 0.001.

^a^Multivariate adjustment for age, sex, visit one year ago, insured amount, urban level, residential area and insured unit.

IR: Incidence density rate = Number of incidence cases/Person-years ∗ 1000.

## Abbreviations

TCM: Traditional Chinese medicine
CAM: Complementary and alternative medicine
LHID2005: Longitudinal Health Insurance Database 2005
LC: Lung cancer
NSCLC: Non-small-cell lung cancer
NHI: National Health Insurance
BNHI: Bureau of National Health Insurance.
